# RO4929097 regulates RANKL-induced osteoclast formation and LPS-mediated bone resorption

**DOI:** 10.18632/aging.202926

**Published:** 2021-05-02

**Authors:** Tao Huang, Congyun Zhao, Yi Zhao, Yuan Zhou, Lei Wang, Donghua Hang

**Affiliations:** 1 Department of Orthopaedics, Baoshan Branch of Shanghai General Hospital, Shanghai Jiao Tong University School of Medicine, Shanghai, China; 2 Department of Orthopaedics, Mang Shi People’s Hospital, Yunnan Province, China; 3 Department of Orthopaedics, Shanghai General Hospital, Shanghai Jiao Tong University School of Medicine, Shanghai, China

**Keywords:** RO4929097, osteoclasts, RANKL, bone resorption, Notch

## Abstract

To investigate the suppressive function of RO4929097, a potent -secretase inhibitor, on RANKL-induced osteoclastogenesis. The cytotoxicity of RO4929097 was evaluated. The suppressive effect and possible molecular mechanism of RO4929097 on RANKL-induced osteoclastogenesis was evaluated both *in vitro* and *in vivo*. The IC50 of RO4929097 was 2.93 μM. Treatment with different doses of RO4929097 (100 nM, 200 nM, and 400 nM) effectively reduced osteoclast formation (number and resorption area) in a dose-dependent manner. The qPCR results revealed that RO4929097 attenuates RANKL-induced osteoclast formation and NFATc1 protein expression. The *in vivo* experiments demonstrated that RO4929097 had an inhibitory effect on LPS-induced bone resorption. Our *in vitro* experiments showed that RO4929097 can potently inhibit osteoclastogenesis and bone resorption by down-regulating the Notch/MAPK/JNK/Akt-mediated reduction of NFATc1. In accordance with these *in vitro* observations, RO4929097 attenuated LPS-induced osteolysis in mice. In conclusion, our findings indicate that Notch may represent a potential therapeutic target for the treatment of osteolytic diseases.

## INTRODUCTION

Bone metabolism is a dynamic process that involves the simultaneous processes of bone resorption and formation [1, 2] Thus, osteoblast and osteoclast disequilibrium can undermine bone integrity and normal functionality, promoting a series of pathological lytic bone disorders [3–5]. Thus, recent research has centered on the investigation and identification of novel agents (synthetic or natural) which may inhibit the pathological formation of osteoclasts and bone resorption [6, 7].

Osteoclasts (OCs) are a type of multinucleated giant cell derived from macrophage precursors. OC differentiation is the first stage of the bone resorptive process, which requires stimulation of receptor activator of NF-κB ligand (RANKL) and macrophage colony stimulating factor (M-CSF). Previous studies have shown that RANKL plays an essential role in the formation of multinucleated OCs [8, 9]. Following an interaction between RANKL and receptor activator of NF-κB (RANK), various downstream signaling pathways (e.g., MAPKs and PI3K/Akt) are activated. These specific pathways are able to upregulate the key regulator of the osteoclast process: nuclear factor of activated T cells c1 (NFATc1) [10–13].

In previous studies, Notch signaling has been shown to play an essential role in multiple developmental pathways [14]. The interaction between Notch receptors and Delta-like1/3/4 (Dlk1/3/4) proteins causes the release of tumor necrosis factor α-converting enzyme (TACE), and γ-secretase [15]. Previous studies have confirmed that the down-regulation of γ-secretase has an inhibitory effect on Notch receptor cleavage. Moreover, Notch has been reported to exhibit both an enhancive and prohibitive effect on RANKL-induced bone resorption [16], suggesting that it may represent a promising targetable pathway for the development of anti-resorptive agents [17, 18]. Furthermore, RO4929097 is a γ-secretase inhibitor that suppresses Notch cleavage; however, its effect on the regulation of osteoclast in the regulation of osteoclast formation has not been confirmed.

In this study, we investigated the effect of RO4929097 on the suppression of RANKL-induced osteoclast formation and elucidated the potential molecular mechanisms associated with this process. We confirmed that RO4929097 suppressed osteoclastogenesis and bone resorption by blocking the Notch/Hes1/MAPK (Erk and p38)/Akt pathway. This conclusion was also confirmed by the *in vivo* results.

## MATERIALS AND METHODS

### BMMs and culture system

BMMs were obtained and cultured as described in previous studies [19–21]. Primary BMMs were isolated from the femurs and tibiae of five-week-old male C57/BL6 mice. All processes were performed under the supervision of Shanghai General Hospital Animal Center Committee of Animal Care and Use (2018371A218). All procedures were conducted in accordance with the guidelines of the Ethical Conduct in the Care and Use of Nonhuman Animals in Research. To select primary BMMs, cells were cultured in complete α-MEM (10% Gibco FBS) containing 33.3 ng/mL M-CSF (R&D Systems, Inc.).

### Cell cytotoxicity assay

The cytotoxicity of RO4929097 (Selleck, Inc.) was determined using an MTT assay. The BMMs were seeded into 96-well plates (1 × 10^4^ cells per well) in complete medium containing 33.3 ng/mL M-CSF and different concentrations of RO4929097 (0, 0.1, 0.2, 0.4, 0.8, 1.6, 3.2, 6.4, 12.8, and 25.6 μM). Following RO4929097 treatment, the cells were incubated for different timepoints (24 h, 48 h, and 96 h). At the end of the process, CCK-8 solution (Dojindo Molecular Technologies, Inc.) was used according to according to the manufacturer’s guidelines.

### Osteoclast formation and TRAP staining assay

The role of RO4929097 on osteoclast formation was assessed. The BMMs were seeded into 96-well plates (1 × 10^4^ cells per well) and stimulated with RANKL (100 ng/mL, R&D Systems, Inc.), M-CSF (33.3 ng/mL), and different concentrations of RO4929097 (0, 100, 200, and 400 nM). The culture medium was replaced every 48 h for six days. After the osteoclasts were observed in the control group, 4% paraformaldehyde was used for OC fixation. TRAP (Sigma-Aldrich; Merck, Inc.) staining solution was added into each of the wells at 37°C for 1 h. TRAP^+^ cells with at least three nuclei were considered to be osteoclasts. Image J software (National Institutes of Health) was used for cell enumeration.

### Bone resorption assay

M-CSF-dependent BMMs were incubated in six-well plates (20 × 10^5^ cells/well). After 24 h, the cells were stimulated with RANKL (100 ng/mL), M-CSF (33.3 ng/mL) until OC formation was observed. The OCs (1 × 10^4^ cells/well ) were then incubated on Corning Osteo Assay Surface plates (Corning, Inc.) and cultured with M-CSF (33.3 ng/mL), RANKL (100 ng/mL), and RO4929097 (0, 100, 200, and 400 nM) for four days. A BioTek Cytation 3 Cell Imaging Reader (BioTek, Winooski, VT) and Image J software were used to photograph and analyze the total resorption pits.

### Immunofluorescence analysis of the podosomal actin belt

OCs derived from BMMs were generated and processed. On days 5–7, pancake-like osteoclasts were observed in the RANKL-treated control group, fixed with 0.1% Triton X-100 (Sigma-Aldrich; Merck&Co, Inc.), and permeabilized for 5 min. After blocking with 1% BSA-PBS for 1 h, the cytoskeletal actin structure was stained with rhodamine-conjugated phalloidin. The immunofluorescence images were obtained using a BioTek Cytation 3 Cell Imaging Reader. Image J software was used to analyze the size (diffusion area) and number of the dental actin bands.

### Real-time PCR analysis

The total RNA (2 × 10^6^ cells) was extracted from the cells using TRIzol^®^ (Invitrogen; Thermo Fisher Scientific, Inc.) according to the manufacturer's protocol. A NanoDrop 2000 spectrophotometer (NanoDrop Technologies; Thermo Fisher Scientific, Inc.) was used to assess the concentration of the total RNA. The following process was performed as described previously. [22] The following mouse primer sets were used in accordance with published reports [21, 23]: mouse NFATc1: forward, 5′-TGCTCCTCCTCCTGCTGCTC-3′ and reverse, 5′-GCAGAAGGTGGAGGTGCAGC-3′; mouse C-fos: forward, 5′-CCAGTCAAGAGCATCAGCAA-3′ and reverse, 5’-AAGTAGTGCAGCCCGGAGTA-3′; mouse Cath-K: forward, 5′-CTTCCAATACGTGCAGCAGA-3′ and reverse, 5′-TCTTCAGGGCTTTCTCGTTC-3′; mouse Trap: forward, 5′-CAAAGAGATCGCCAGAACCG-3′ and reverse, 5′-GAGACGTTGCCAAGGTGATC-3’; mouse GAPDH: forward, 5′-CACCATGGGA GAAGGCCGGGG-3′ and reverse, 3′-GACGGACACA TTGGGGGTAG-5′.

### Western blotting analysis

The total cellular proteins (TCPs) were extracted at different time points. Following treatment with 400 nM RO4929097 for 2 h, RANKL (100 ng/mL) was used to stimulate the cells for different periods of time (short time course). The membrane was blocked in 1% TBST (tri-buffered saline, Tween 20) in 5% skim milk at room temperature for 1 h, and subsequently reacted with primary antibodies NFATc1 (cat no. 5861S; 1:1,000; Cell Signaling Technology, Inc.); Cleaved Notch1 (cat no. 4147S; 1:1,000; Cell Signaling Technology, Inc.); HES1 (cat no.11988; 1:1,000; Cell Signaling Technology, Inc.); GAPDH (cat no.174S; 1:1,000; Cell Signaling Technology, Inc.); p-Akt (cat no.4060S; 1:1,000; Cell Signaling Technology, Inc.); Akt (cat no.4685S; 1:1,000; Cell Signaling Technology, Inc.); p-ERK (cat no.4370S; 1:1,000; Cell Signaling Technology, Inc.); Erk (cat no.9194S; 1:1,000; Cell Signaling Technology, Inc.); p65 (cat no.8242S; 1:1,000; Cell Signaling Technology, Inc.); p-p38 (cat no.4511; 1:1,000; Cell Signaling Technology, Inc.); p38 (cat no.8690; 1:1,000; Cell Signaling Technology, Inc.); p-p65 (cat no.3033; 1:1,000; Cell Signaling Technology, Inc.). After incubating the samples overnight at 4°C, they were incubated with a secondary antibody at room temperature for 1 h using Odyssey V3 .0 image scan (Li-COR. Inc., Lincoln, NE, USA) to observe antibody reactivity.

### Mouse model of LPS-Induced calvarial bone resorption

All processes were was carried out in compliance with the ARRIVE guidelines and under the supervision of Shanghai General Hospital Animal Center Committee of Animal Care and Use. All procedures were performed under the guidelines of the Ethical Conduct in the Care and Use of Nonhuman Animals in Research. Male C57BL/6 mice (aged 7 to 8 weeks), were randomly assigned to four groups: Control (PBS), LPS and LPS with low (400 nM) or high (800 nM) concentrations of RO4929097. A 1% pentobarbital sodium (10 mg/kg) was injected intraperitoneally to anaesthetize the mice. The knees of the mice were locally injected with 100 μL LPS or PBS (10 mg/kg), and with or without RO4929097, respectively, for two weeks. During the experiment period, no significant adverse or toxic effects were observed. The mice did not exhibit significant weight loss or lack of spirit, and none of the mice died during the experimental period. Then, euthanasia was performed using pentobarbital sodium (100 mg/kg) [24]. The knee joints were resected and fixed in 4% paraformaldehyde for 48 h. Micro-computed tomography (CT) scanning was performed using high-resolution micro-CT (μCT-100, SCANCO Medical AG, Switzerland). The resolution of the scanning was 10 μm; the X-ray energy was set at 70 kv, 200 μA; and the fixed exposure time was 300 ms. We defined the region of interest (ROI) to encompass the entire subchondral bone in the tibial plateaus.

### Histological staining and histomorphometric analysis

The PFA-fixed mice knees were decalcified in 10% EDTA for two weeks and embedded in paraffin. Next, the samples were sliced into specific sections (4 μm thick) and subjected to H&E and TRAP staining. Digital images were obtained using an Axio ScopeA1 light microscope (ZEISS, Inc.). The osteoclasts were enumerated using Image J software.

### Statistical analysis

All data were presented as the mean ± standard deviation (SD). A Student's *t*-test was used to assess the differences between the control and therapeutic groups. The results for the multiple group comparisons were analyzed using a Scheffe's test and one-way analysis of variance (ANOVA) with SPSS 22.0 software (SPSS, Inc.). Values were determined to be significant at ^*^P < 0.05; ^**^P < 0.01; and ^***^P < 0.001.

### Availability of data and materials

The datasets supporting the conclusions of this article are available in an open access repository.

### Ethics approval and consent to participate

The study design was carried out in compliance with the ARRIVE guidelines and approved by the Ethical Committee of Shanghai General Hospital.

## RESULTS

### RANKL-induced osteoclast formation *in vitro* was down-regulated by RO4929097

**Figure 1:**
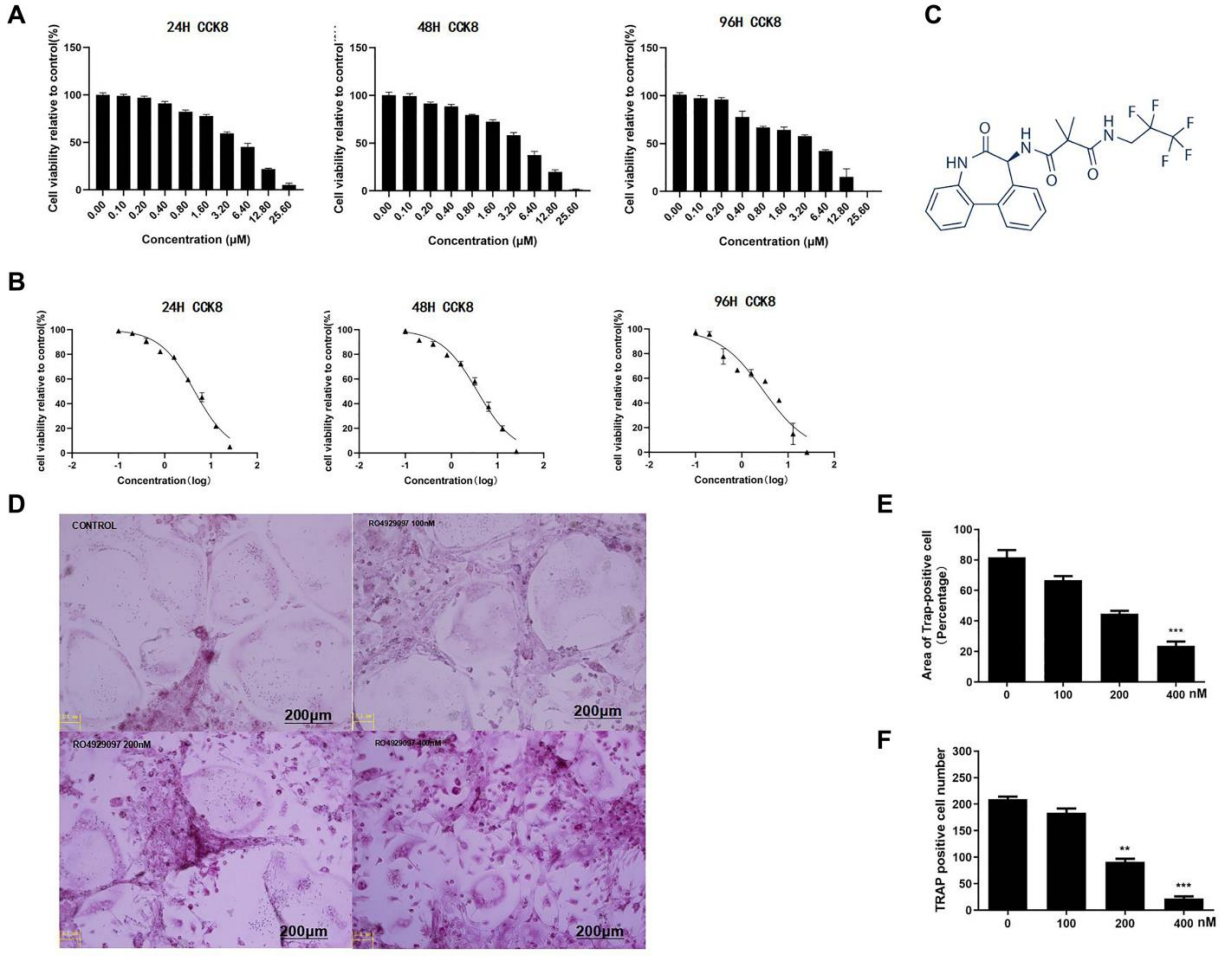
**RANKL-Induced osteoclast formation *in vitro* was down-regulated by RO4929097.** (**A**) RO4929097cytotoxicity. (**B**) The results showed that the IC50 of RO4929097 at different time points in the BMMs was 4.44 μM, 3.66 μM, and 2.93 μM, respectively. (**C**) The chemical structural formula of RO4929097 (provided by Selleck). (**D**) BMMs were simulated with different concentrations of RO4929097 combined with M-CSF (33.3 ng/mL) and RANKL (100 ng/mL) for five days. Cells were fixed in 4% paraformaldehyde and stained for TRAP. (**E** and **F**) The area and number of TRAP-positive cells. The data were presented as the mean ± SD (^*^p < 0.05, ^**^p < 0.01, ^***^p < 0.001).

First, the cytotoxicity of RO4929097 was evaluated. The results showed that the IC50 of RO4929097 in the BMMs at 24 h, 48 h, and 96 h was 4.44 μM, 3.66 μM, and 2.93 μM respectively (Figure 1A and 1B). Figure 1C presents the chemical structure formula of RO4929097 (provided by Selleck). Compared with the control group, RO4929097 treatment could effectively reduce the number and area of OCs in a dose-dependent manner (Figure 1D, 1E and 1F). Accordingly, RO4929097 was able to inhibit osteoclast formation at a specific concentration under IC50.

### Podosome actin belt formation and OC-mediated bone resorption activity was inhibited by RO4929097

**Figure 2:**
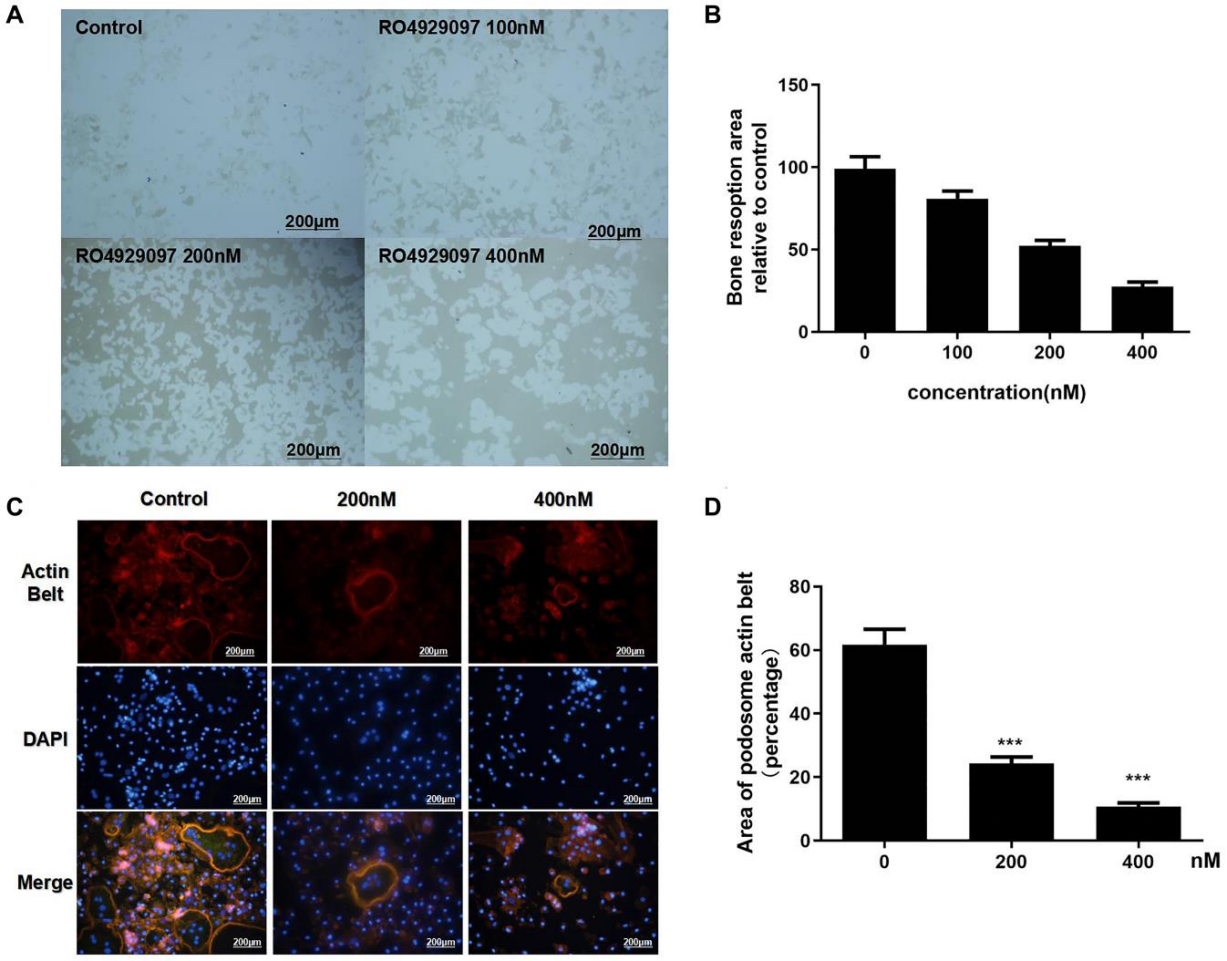
**Podosome actin belt formation and OC-mediated bone resorption activity was inhibited by RO4929097.** (**A**) OCL were seeded onto Osteo Assay Stripwell Plates with RANKL (100 ng/mL), M-CSF (33.3 ng/mL), and RO4929097 (0,100, 200, and 400 nM) for four days (**B**) The resorption area in Osteo Assay Stripwell Plates. (**C**) BMMs were cultured in 48-well plates with 0, 100, 200, and 400 nM RO4929097 for five days. The cells were fixed and stained for immunofluorescence. (**D**) Area of OCs with an F-actin belt. The data were presented as the mean ± SD.

To assess the effect of RO4929097 on OC-mediated bone resorption activity, the resorption area on the hydroxyapatite-coated Osteo Assay plates was calculated (Figure 2A). In accordance with the observed osteoclast reduction, the bone resorption area was also reduced in a dose-dependent manner (Figure 2B). The immunofluorescence results showed that podosome actin belt formation was suppressed by RO4929097 in a dose-dependent manner (Figure 2C). In summary, these data show that RO4929097 could effectively inhibit podosome actin belt formation (Figure 2D).

### RO4929097 suppressed the relative expression of osteoclastogenesis genes

**Figure 3:**
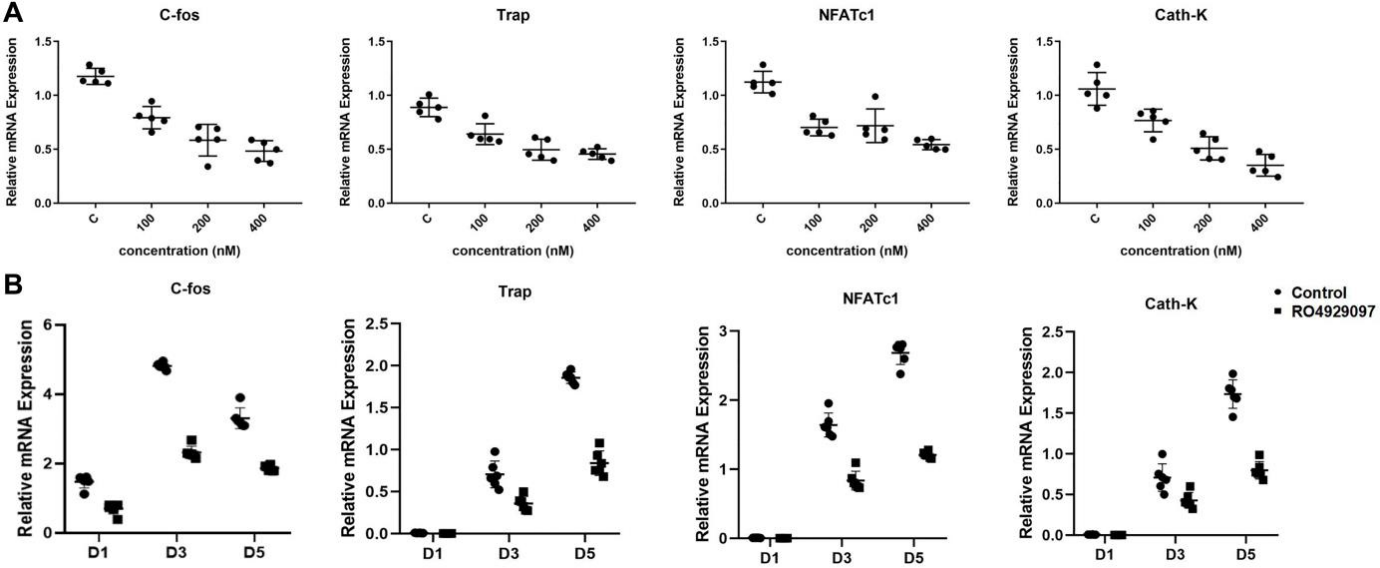
**RO4929097 suppressed the relative expression of osteoclastogenesis genes.** (**A**) BMMs were treated with M-CSF (33.3 ng/mL), RANKL (100 ng/mL) in the presence of 0, 100, 200, and 400 nM RO4929097 for five days. The expression of the osteoclast-specific genes, including NFATc1, Cath-K, TRAP, and C-fos were analyzed using quantitative real-time PCR. (**B**) The BMMs were treated with M-CSF (33.3 ng/mL) and RANKL (100 ng/mL) in the presence 400 nM RO4929097 for 1, 3, and 5 days. Osteoclast-specific gene expression was analyzed using quantitative real-time PCR. RNA expression levels were normalized to the expression of GAPDH. The data were presented as the mean ± SD (^*^p < 0.05; ^**^p < 0.01; ^***^p < 0.001).

Quantitative PCR (qPCR) was used to evaluate the relative expression of osteoclastogenesis genes at the mRNA level. It was observed that the relative gene expression of NFATc1, C-fos, Cath-K, and TRAP was markedly downregulated by RO4929097 in both a dose-dependent and time-dependent manner. (Figure 3A and 3B).

### RO4929097 mediates osteoclastogenesis by inhibiting Notch, MAPK, and Akt signaling in BMMs

**Figure 4:**
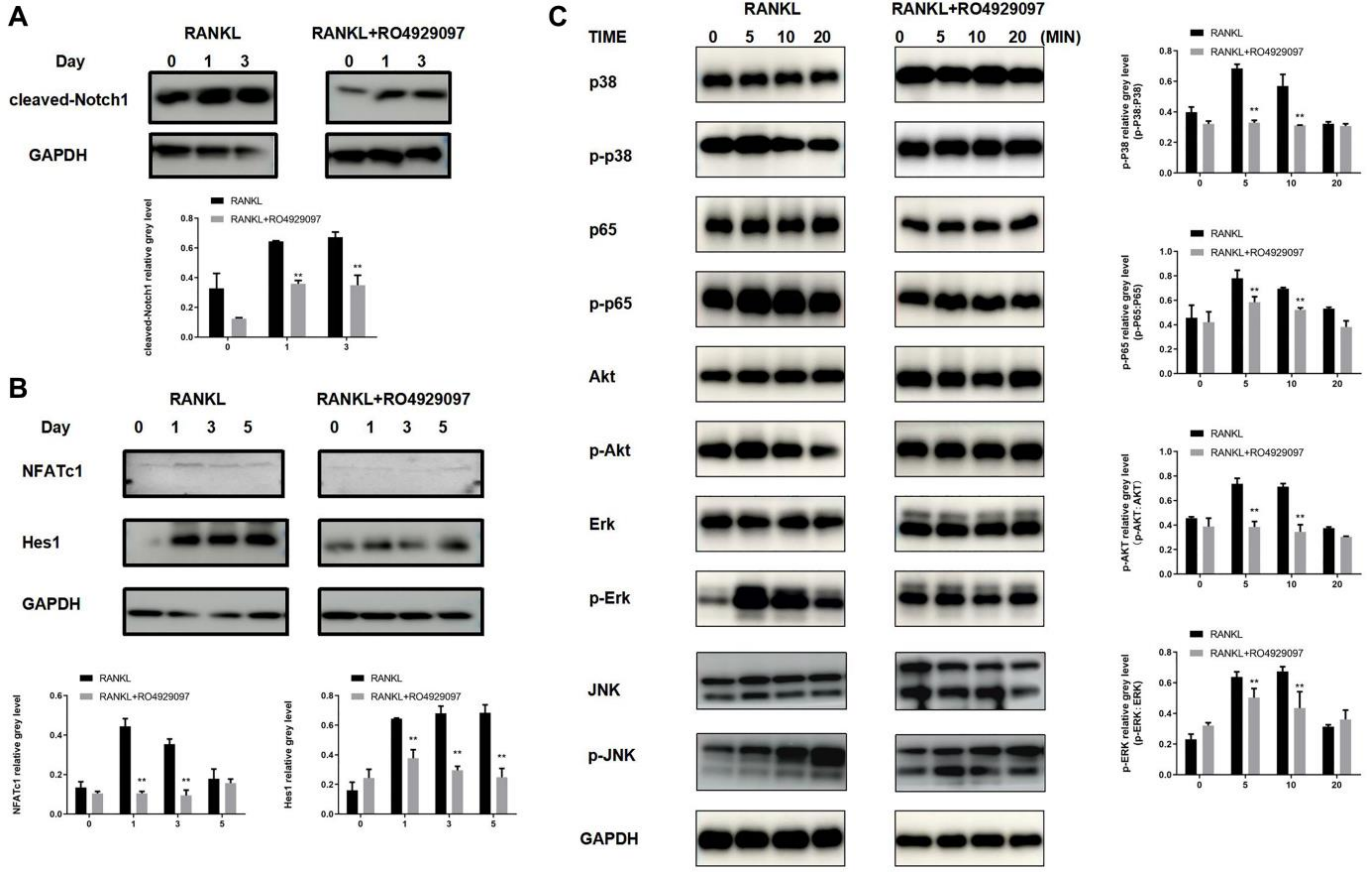
**RO4929097 suppresses osteoclastogenesis via Notch inhibition and the regulation of AKT phosphorylation.** (**A**) RO4929097 treatment suppressed the expression of cleaved Notch1. (**B**) RO4929097 treatment suppressed the protein expression of Hes1 and NFATc1. (**C**) RO4929097 treatment suppressed the expression of AKT phosphorylation. The data were presented as the mean ± SD (^*^p < 0.05; ^**^p < 0.01; ^***^p < 0.001).

To investigate the potential molecular mechanisms of RO4929097 in the inhibition osteoclastogenesis, the RANKL-induced NF-κB, MAPK, JNK and PI3K/Akt pathways were examined using a Western blot analysis. BMM treatment with RO4929097 during RANKL-induced osteoclast formation induced cleaved Notch1, Hes1, and NFATc1 protein expression (Figure 4A and 4B). In addition, short-term stimulation with RO4929097 significantly reduced the phosphorylation of p38, ERK1/2, p65, JNK and Akt (Figure 4C). Accordingly, our results suggest that RO4929097 inhibited osteoclastogenesis by preventing Notch receptor cleavage and Notch signaling, as well as by perturbing the activation of the ERK1/2, p38, JNK and AKT signaling cascades.

### RO4929097 administration prevents LPS-induced bone resorption *in vivo*

**Figure 5:**
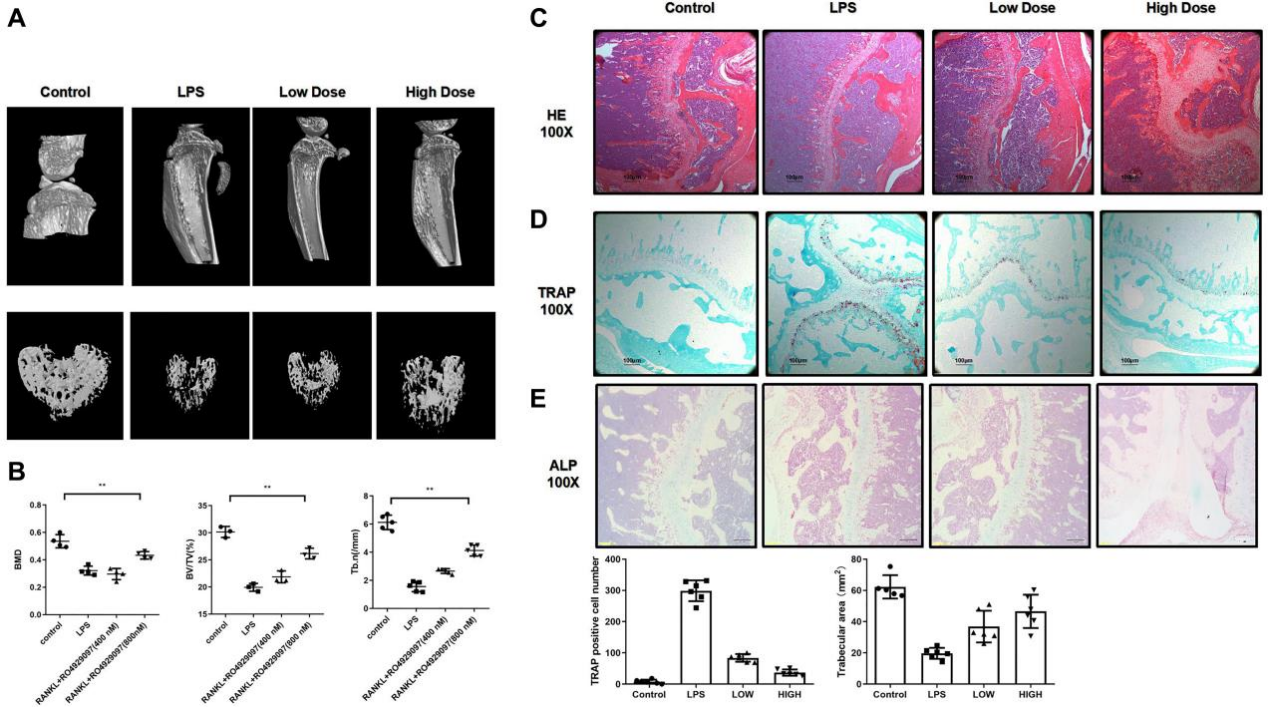
**RO4929097 attenuated lipopolysaccharide-induced bone resorption in mouse knees *in vivo*.** (**A**) Images of a three dimension reconstruction based on micro-CT scanning. (**B**) Results of BMD, bone BV/TV and Tb.n. (**C**, **D** and **E**) HE TRAP and ALP results. Amount of TRAP-positive OCs. The data are presented as the mean ± SD (^*^p < 0.05; ^**^p < 0.01; ^***^p < 0.001).

The *in vitro* experiments were used to investigate the effect of RO4929097 on RANKL-induced osteoclastogenesis and bone resorption function by studying the phenotype and associated mechanism. We investigated the function of RO4929097 in mice with LPS-induced bone resorption. According to the three-dimensional micro-CT reconstruction shown in Figure 5A, compared with the control group, the RO4929097-treated groups displayed fewer and smaller resorption pits. A significant reduction in BV/TV, Tb.n, and BMD was also observed in the LPS group (Figure 5B).

The histological analysis also revealed an inhibitory effect of RO4929097 on LPS-induced bone resorption *in vivo*. In accordance with previous results, extensive osteolysis was observed in the HE-stained images of the control group, whereas a lower osteolytic level was observed in the RO4929097-treated groups (Figure 5C). The TRAP staining results confirmed a decreased number of OCs following RO4929097 treatment (Figure 5D). No difference was observed in the ALP staining under RO4929097 treatment (Figure 5E). Accordingly, the *in vivo* results confirmed the potential therapeutic application of RO4929097 in inflammation-relative osteolytic disease.

## DISCUSSION

Pathological bone homeostasis promotes the over-formation and activation of OCs, leading to substantial bone disruption, which is a feature of several osteolytic diseases [3–5]. Medical therapies that suppress osteolysis are considered to be a potential treatment strategy for these diseases [25, 26]. However, several of these treatments are associated with various side-effects [27, 28]. Accordingly, the development of medications that can inhibit osteoclastogenesis without side-effects are urgently required.

Our research reveals potential signaling pathways which may regulate RANKL-induced osteoclast formation. Notch signaling is important in many cellular functions, including morphogenesis and stem cell niche maintenance [29–31]. However, the function of Notch signaling on osteoclastogenesis remains unclear, with evidence supporting both a stimulatory and inhibitory role. According to previous research, Notch has been shown to act in conjunction with MAPK, NF-κB, and PI3K/Akt to potentially regulate NFATc1 expression during osteoclast differentiation [17, 32–35]. In addition, Notch1 suppression in BMMs has been confirmed to promote osteoclastogenesis *in vitro* [36], whereas Notch2 suppression attenuates osteoclastogenesis [16, 37].

Previous research has confirmed a synergistic function between Notch and NF-κB signaling [38]. Our findings indicate that NFATc1 expression was significantly decreased in response to RO4929097 treatment. As a potent γ-secretase inhibitor, RO4929097 does not alter the level of Notch expression but rather blocked its cleavage. Accordingly, the expression of cleaved Notch1 (Notch intracellular domain-1; NICD1) and hairy enhancer of split 1 (HES1), a targeted gene regulated by cleaved Notch1, were evaluated were evaluated in BMMS stimulated for longer time points. In accordance with previous studies [39], we also observed a reduction in the level of ERK, p38, Akt, and p65 phosphorylation in response to RO4929097 treatment. This indicates that there is significant crosstalk between Notch, ERK, and Akt signaling. Our data do suggest that RO4929097 can suppress bone loss and osteoclast formation via Notch-mediated NFATc1 expression.

In this study, a widely-accepted LPS-induced osteolytic experimental model was established to assess the effect of RO4929097. During the experimental design for this study, Ovx, RANKL-induced bone resorption, and LPS- induced bone resorption models were considered. The Ovx model was considered to be inappropriate for this research because Ovx regulates both osteoclast and osteoblast formation. The reason RANKL is used for *in vitro* studies is because RANKL is mainly produced by osteoblasts and is necessary for the formation of osteoclasts; however, osteoblasts are present *in vivo*. LPS-induced bone resorption, as a general approach, has been widely used for osteoclast-related *in vivo* studies. [40–44] Thus, LPS-induced bone resorption was selected as the *in vivo* model in this study. Both the micro-CT analysis and histological staining of TRAP confirmed downregulated osteopenia in the RO4929097-treated groups. However, the present study was associated with some limitations. First, our study did not address the possibility that RO4929097 regulates osteoclasts via other signaling pathways. Second, the influence of RO4929097 on osteoblast and bone formation should be further assessed.

In conclusion, our data demonstrate that RO4929097 can effectively attenuate RANKL-induced osteoclastogenesis *in vitro* via downregulating Notch/MAPK (ERK and p38)/Akt signaling. In accordance with the *in vitro* observations, prevention of LPS-induced osteolysis *in vivo* also confirmed that RO4929097 may represent a potential treatment strategy for osteolytic diseases in relation to excessive osteoclast-mediated bone resorption.
